# In Vitro Sensitivity of *Plasmodium falciparum* from China-Myanmar Border Area to Major ACT Drugs and Polymorphisms in Potential Target Genes

**DOI:** 10.1371/journal.pone.0030927

**Published:** 2012-05-31

**Authors:** Zenglei Wang, Daniel Parker, Hao Meng, Lanou Wu, Jia Li, Zhen Zhao, Rongping Zhang, Qi Fan, Haiyan Wang, Liwang Cui, Zhaoqing Yang

**Affiliations:** 1 Department of Entomology, Pennsylvania State University, University Park, Pennsylvania, United States of America; 2 Department of Parasitology, Kunming Medical University, Kunming, Yunnan, China; 3 Department of Pharmacology, Kunming Medical University, Kunming, Yunnan, China; 4 Department of Pharmaceutical Chemistry, Kunming Medical University, Kunming, Yunnan, China; 5 Dalian Institute of Biotechnology, Dalian, Liaoning, China; 6 Department of Statistics, Kansas State University, Manhattan, Kansas, United States of America; University of Oklahoma Health Sciences Center, United States of America

## Abstract

Drug resistance has always been one of the most important impediments to global malaria control. Artemisinin resistance has recently been confirmed in the Greater Mekong Subregion (GMS) and efforts for surveillance and containment are intensified. To determine potential mechanisms of artemisinin resistance and monitor the emergence and spread of resistance in other regions of the GMS, we investigated the *in vitro* sensitivity of 51 culture-adapted parasite isolates from the China-Myanmar border area to four drugs. The 50% inhibitory concentrations (IC_50_s) of dihydroartemisinin, mefloquine and lumefantrine were clustered in a relatively narrow, 3- to 6-fold range, whereas the IC_50_ range of artesunate was 12-fold. We assessed the polymorphisms of candidate resistance genes *pfcrt*, *pfmdr1*, *pfATP6*, *pfmdr6* and *pfMT* (a putative metabolite/drug transporter). The K76T mutation in pfcrt reached fixation in the study parasite population, whereas point mutations in pfmdr1 and pfATP6 had low levels of prevalence. In addition, *pfmdr1* gene amplification was not detected. None of the mutations in pfmdr1 and pfATP6 was associated significantly with *in vitro* sensitivity to artemisinin derivatives. The ABC transporter gene *pfmdr6* harbored two point mutations, two indels, and number variations in three simple repeats. Only the length variation in a microsatellite repeat appeared associated with altered sensitivity to dihydroartemisinin. The *PfMT* gene had two point mutations and one codon deletion; the I30N and N496– both reached high levels of prevalence. However, none of the SNPs or haplotypes in *PfMT* were correlated significantly with resistance to the four tested drugs. Compared with other parasite populations from the GMS, our studies revealed drastically different genotype and drug sensitivity profiles in parasites from the China-Myanmar border area, where artemisinins have been deployed extensively for over 30 years.

## Introduction

The development and spread of multidrug resistant (MDR) *Plasmodium falciparum* has led to the adoption of artemisinin-based combination therapies (ACTs) as the first-line treatment for falciparum malaria in most malaria-endemic countries of the world [1]. Artemisinin and its derivatives are by far the most potent antimalarial drugs [2], and at present, our last line of defense against multidrug resistant parasites. Therefore, the recently confirmed emergence of artemisinin resistance in western Cambodia is a major threat for current initiatives to control and eliminate malaria [3]–[5]. Because this exact same area has been the origin of both chloroquine (CQ) and sulfadoxine-pyrimethamine resistance, both of which have subsequently spread to Africa [6], [7], the consequence of a similar spread of artemisinin resistance will be catastrophic. While limited evidence suggests that artemisinin resistance has not yet spread to other areas [8], the World Health Organization (WHO) is coordinating a large-scale elimination campaign in this region aiming to contain artemisinin resistance [9], [10]. Apparently, containment efforts require better resistance surveillance [11], but this is hampered due to the lack of convenient molecular surveillance tools for detecting artemisinin resistance. At this moment, the most reliable way of detecting artemisinin resistance is through rigorous clinical efficacy studies, which are expensive and time-consuming.

The mode of action of artemisinins in malaria parasites is still a debated topic and the molecular basis of reduced artemisinin susceptibility is unclear [12]–[14]. To date, a few genes have been postulated to be associated with artemisinin resistance. The *P. falciparum multiple drug resistance 1* (*pfmdr1*) gene has received the most attention, beacuse several mutations (N86Y, Y184F, S1034C, N1042D, and D1246Y) occurring in PfMDR1 from field isolates are associated with altered sensitivity to multiple structurally unrelated antimalarials such as CQ, mefloquine (MQ), quinine (QN), halofantrine (HF), and artemisinins [15]–[19]. In addition, *pfmdr1* amplification is a key determinant for both *in vivo* and *in vitro* resistance to MQ and HF [19]–[23]. Increased *pfmdr1* copy number, which is more prevalent in west Cambodia, is associated with increased risk of therapy failures of artesunate (AS)-MQ, the major ACT deployed in Thailand and Cambodia [24]–[28]. The sarco/endoplasmic reticulum calcium-dependent ATPase (SERCA) homologue PfATP6 has been considered as a specific target of artemisinins, since artemisinin derivatives inhibit this enzyme expressed in *Xenopus* oocytes [29] and this inhibition was abolished by the introduction of the L263E mutation in the predicted transmembrane domain 3 of PfATP6 [30]. Another mutation (S769N) has been linked to artemether resistance in *P. falciparum* field isolates from French Guiana [31]. However, the L263E mutation has not been found in field isolates from most malaria endemic areas, and S769N is very rare [32]–[40]. Though multiple new single nucleotide polymorphisms (SNPs) have been detected in *pfatp6*, their associations with artemisinin resistance have not been established. Mutations in several other genes also have been suggested to be responsible for artemisinin resistance. Mutations in the *UBP1* gene encoding a deubiquitination enzyme have been identified to confer artemisinin resistance in the rodent malaria parasite *P. chabaudi* [41]. However, the equivalent mutations have not been found in *P. falciparum* field isolates from Cambodia and Thailand [42]. Recently, using a genome-wide association approach, Mu et al. detected signs of positive selection at several putative transporter genes in parasite populations including one coding the ABC transporter pfmdr6 and another gene coding the metabolite/drug transporter pfMT [43], [44]. So far, none of the candidate genes have been conclusively shown to be responsible for artemisinin resistance. Therefore, further research is needed to identify the causal mutations for artemisinin resistance.

Malaria is still a serious public health problem in the Greater Mekong Subregion (GMS), which includes Cambodia, Laos, Thailand, Vietnam, Myanmar and China [45]. Within this region, malaria transmission is particularly intense along international border areas. The GMS is historically a hotspot of antimalarial drug resistance, and resistance management remains a high priority for malaria control. The China-Myanmar border area has the longest history of artemisinin monotherapy; it has been used for over three decades and *in vitro* studies have detected reduced susceptibility to artemisinins [46]. Therefore, close surveillance of artemisinin resistance in this area is necessary to detect and deter resistance development. Here we report an assessment of *in vitro* sensitivity of clinical *P. falciparum* isolates from the China-Myanmar border area to four antimalarial drugs, AS, dihydroartemisinin (DHA), lumefantrine (LMF) and MQ. Using a candidate gene approach, we want to determine whether polymorphisms in these genes are associated with decreased drug sensitivity.

## Materials and Methods

### Parasite Samples and *in vitro* Culture

*P. falciparum* clinical isolates were collected in 2007–2009 from symptomatic patients presenting with uncomplicated *P. falciparum* infections attending a malaria clinic in Laiza township near the China-Myanmar border. Malaria infections were diagnosed by microscopic examination of Giemsa-stained thick and thin blood films. To confirm monoclonal infections, parasites were genotyped at three polymorphic genes, *merozoite surface protein 1* (*msp1*), *msp2* and *glutamate-rich protein* (*glurp*), using previously described methods [47]–[49]. A total of 21, 9, and 21 parasite isolates were adapted to continuous *in vitro* culture for the year 2007, 2008, and 2009, respectively [50]. The human subject protocol for this study was approved by the Institutional Review Board of Kunming Medical University. Parasite routine culture was maintained in type O^+^ human red blood cells (RBCs) in complete medium supplemented with 6% AB human serum under an atmosphere of 90% N_2_/5% O_2_/5% CO_2_ [51].

### *In vitro* Drug Sensitivity Assay

A SYBR Green I-based fluorescence assay was used to measure parasite sensitivity to four antimalarial drugs: AS, DHA, MQ, and LMF [52]. MQ was purchased from Sigma (St Louis, MO, USA), while the other three drugs were obtained from Kunming Pharmaceutical Co. (Kunming, Yunnan, China). The stock solution of AS (0.260 mM), DHA (0.352 mM), MQ (2.56 mM), and LMF (7.56 mM) were prepared in ethanol and 2-fold dilutions were made in complete medium. Cultures were synchronized by two rounds of sorbitol treatment, and late ring or early trophozoite stage parasites were assayed for drug sensitivity in 96-well microtiter plates at 5% hematocrit and 0.3% parasitemia. For drug assays, 90 µl of parasite culture were seeded into each culture well, and 10 µl of diluted drugs in complete medium solutions were dispensed into each well to obtain a desired final concentration. The plates were incubated at 37°C in a CO_2_ incubator for 72 h. The plates were then frozen at −80°C, thawed, and mixed with 100 µl of lysis buffer. The plates were incubated in the dark for about 1 h and fluorescence data were acquired using the Fluoroskan Ascent FL microplate fluorometer (Thermo Scientific, Waltham, MA). For each parasite isolate and drug concentration, the assay was performed in three biological replicates and each with three technical replicates. To reduce the variations between plates, the standard laboratory clone 3D7 was included as a reference. The *in vitro* drug response data were entered into the SPSS data editor, and the geometric mean of the half-maximal inhibitory concentration (IC_50_) was calculated for all isolates using a regression-probit analysis. Standard deviation was calculated using the mean values of the three biological replicates of each parasite isolate.

### Molecular Analysis of Candidate Genes

Parasite genomic DNA was isolated from cultured parasites using a proteinase K digestion and phenol/chloroform extraction procedure [53]. By using a PCR and sequencing approach, we assessed SNPs in *pfcrt*, *pfmdr1*, *pfatp6,* a putative ABC transporter *PF13_0271* (*pfmdr6*), and a putative metabolite/drug transporter *PF14_0260* (*pfMT*). Two *pfcrt* fragments covering codons 76 and 220 and two *pfmdr1* fragments including codons 86, 184, 1034, 1042 and 1246 were amplified as described previously [54]. Two regions of *pfatp6* gene (28–3065 and 3207–3952 bp) were amplified using primers and conditions from previously published work [36], [55] (see Table S1). The coding sequences of *pfmdr6* and *pfMT* genes, shown to be under strong positive selection [43], were amplified using primers shown in Table S1. To minimize errors introduced in the sequences during PCR amplification, we used Advantage HD DNA Polymerase Mix (Clontech, Mountain View, CA), which has efficient 3′ → 5′ exonuclease activity for high fidelity. PCR products were purified using a PCR Purification kit (Qiagen, Valencia, CA) and used for direct sequencing. Overlapping sequences were obtained by using sequencing primers. For singletons, the sequence was confirmed from two independent PCR reactions from the same DNA templates. For a highly AT-rich region in the *pfMT* gene, the primers MT_873 and mt_1433R were used to amplify this fragment and the PCR products were cloned into the pGEM-T Easy Vectors system (Promega, Madison, WI). Plasmid DNA was extracted by Zyppy Plasmid Miniprep Kit (Zymo research, Orange, CA). For accuracy, at least two clones were sequenced for each sample. Alignment of DNA sequences were performed using the BioEdit program with the 3D7 sequence as the reference. All new sequences were deposited in GenBank under the accession numbers (JN983240–JN983290 for *pfatp6*, JN983291–JN983341for *pfmdr6*, and JN983342–JN983392 for *pfMT*).

*Pfmdr1* copy number in field parasite isolates was determined by real-time PCR using FastStart Universal SYBR Green Master Mix on an ABI 7300 real-time PCR system (Applied Biosystems). DNA from the reference strain 3D7, which has a single copy *pfmdr1* gene, was included for calibration. The single copy *β-tubulin* gene served as an internal control for estimating the copy number of *pfmdr1*. Each reaction was performed in a final reaction volume of 20 µl. Copy number of *pfmdr1* gene was calculated using the 2^−ΔΔ*Ct*^ method as described earlier [56]. The efficiency of each PCR (*pfmdr1* and *β-tubulin*) was determined using a scale dilution of DNAs from the laboratory lines 3D7 (containing 1 copy of *pfmdr1*) and Dd2 (containing 3–4 copies of *pfmdr1*). Determination of the copy number was done by comparison of the ratio of *pfmdr1*/*β-tubulin* taking into account the efficiency of each PCR. Each sample was analyzed in triplicate. Assays were repeated if one of the following two results was obtained: *C_t_* value >35, or copy number as 1.3–1.6 or <0.7.

### Statistical Analysis

Spearman’s correlation coefficients were used to investigate the degree and significance of the relation between the IC_50_ values of the four drugs. It was chosen instead of Pearson’s in order to provide robust correlation estimates despite potential outliers in the data. We then used *t*-tests to look for significant differences in mean IC_50_ values between these field isolates and 3D7, and between the years. Finally, we used multiple *t*-tests and a generalized linear model to investigate potential, significant differences in mean IC_50_ values between haplotypes and point mutations for each gene and for each drug. The Bonferroni correction was used to control for the increased probability of false positives in multiple comparisons. All statistical analyses were done using SAS version 9.2 (http://www.sas.com/) and R version 2.14.2 (http://cran.r-project.org/).

## Results

### *In vitro* Drug Assays

**Figure 1:**
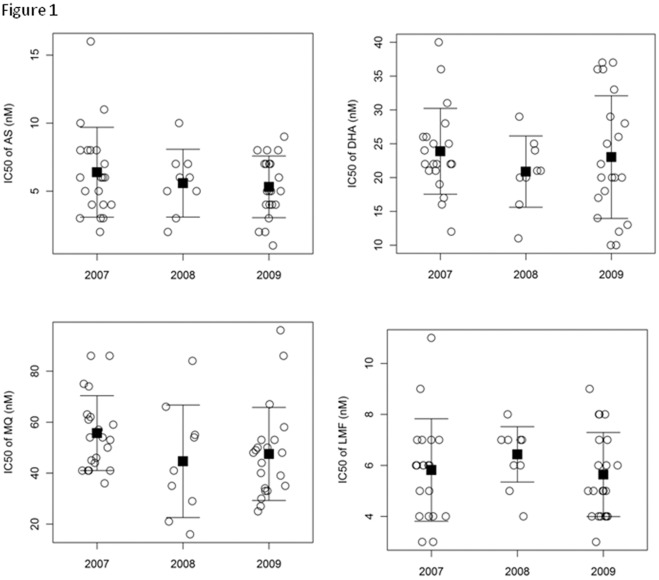
*In vitro* drug responses. IC50s (nM) of P. falciparum isolates to artesunate (AS), dihydroartemisinin (DHA), mefloquine (MQ) and lumefantrine (LMF) are stratified by year. Solid boxes (▪) indicate the mean values; the error bars denote one standard deviation. For IC_50_ values of each drug, there was no significant differences between the years (*P*>0.05, *t-*test).

To determine whether artemisinin resistance has emerged at the China-Myanmar border area, we assessed *in vitro* sensitivity of 51 culture-adapted *P. falciparum* strains originating from this region to two artemisinin derivatives AS and DHA and two aminoalcoholic drugs MQ and LMF. These parasites were collected in 2007–2009 and genotyped to confirm monoclonal infections [54]. The IC_50_ values of individual parasite isolates are shown in Table S2. Parasite isolates were generally sensitive to AS with a mean IC_50_ of 5.8 nM, but the range (1.4–16.5 nM) was relatively wide with an almost 12-fold difference between the most and least susceptible isolates (Table 1). The mean IC_50_s of the samples to DHA ranged from 9.6 to 40.3 nM with a mean IC_50_ of 23.0 nM. The mean IC_50_ for MQ was 50.5 nM, ranging from 16.2 to 96.2 nM, with a six-fold difference in IC_50_ between the most and the least susceptible isolates. The parasites had a mean IC_50_ of 5.9 nM for LMF with a fairly narrow range of 3.0–11.5 nM. Compared with the mean values of the control laboratory clone 3D7, only MQ sensitivity showed a significant difference after Bonferroni correction (*P<*0.05). When the IC_50_ data were stratified by years, slight variations were observed between the years (Figure 1). However, none of the comparisons were statistically significant (*P*>0.05, *t*-test). Also noteworthy is the detection of several outlier isolates (greater than 2 standard deviation), which showed much higher IC_50_ values to all drugs.

**Table 1:** *In vitro* IC_50_s (nM) of 3D7 and 51 culture-adapted clinical isolates to four antimalarial drugs.

Drug	3D7(Mean ± SD)	Clinical isolates	
		Mean ± SD	Range
Artesunate	5.4±1.5	5.8±2.8	1.4–16.5
Dihydroartemisinin	22.1±2.0	23.0±7.4	9.6–40.4
Mefloquine	40.7±11.7	50.4±17.8*	16.2–96.2
Lumefantrine	7.12±2.1	5.9±1.7	3.0–11.5

IC_50_ values are shown as mean ± standard deviation (SD). The mean IC_50_ of each clinical isolate was used to calculate the mean IC_50_ and SD for all isolates. The range of IC_50_ values of all clinical isolates is also shown for each drug. *t*-tests were used to compare the IC_50_ values between test samples and laboratory clone 3D7. Asterisk (*) indicates statistically significant difference from 3D7 after a Bonferroni correction (*P*<0.0009).

Correlations between the IC_50_s of the four antimalarial drugs were evaluated using the Spearman’s test (Table 2). There was a highly significant correlation between responses to the two artemisinin derivatives (*P*<0.0001). When all samples were considered, we did not detect significant correlation between the two aminoalcohol drugs MQ and LMF (*P*>0.05). However, when only the samples collected in 2009 were compared, a modest correlation was found between MQ and LMF (*P*<0.05) (data not shown).

**Table 2:** Correlation matrix showing Spearman’s correlation coefficients between the four tested drugs.

	Mefloquine	Lumefantrine	Dihydroartemisinin	Artesunate
Mefloquine	−			
Lumefantrine	0.2060	−		
Dihydroartemisinin	−0.0056	0.2316	−	
Artesunate	0.2523	0.1991	0.5494*	−

* indicates *P*≤0.0001.

### Polymorphisms in Candidate Target Genes

#### Pfmdr1

We genotyped *pfmdr1*SNPs by PCR and sequencing in the 51 field isolates, focusing on the known amino acid substitutions at codons 86 (N/Y), 184 (Y/F), 1034 (S/C), 1042 (N/D), and 1246 (D/Y). Wild-type haplotype was prevalent and accounted for ∼65%. Except codon 184, mutations at other codons were rare (Table 3). Specifically, mutations at codons 1034 and 1246 were not observed. The N86Y mutation, which is associated with CQ resistance and increased sensitivity to MQ and artemisinin [57], was detected only in one (2%) isolate of the samples. The N1042D mutation associated with QN resistance was also rare and found only in 4 (7.8%) isolates of the samples [18]. The C-terminal mutation haplotype 1034C/1042D/1246Y, which is prevalent in South America and enhances parasite susceptibility to MQ and artemisinin, was absent in our samples (Table 3). We did not detect *pfmdr1* amplification in any samples; the 51 isolates had a mean *pfmdr1* copy number value of 1.02±0.14 (Figure S1).

**Table 3:** Amino acid substitutions and resulting haplotypes in four studied genes from 51 parasite isolates.

Gene	Mutation	No. (%)	Haplotypes	No. (%)
Pfmdr1(86/184/1042)	N86**Y**	1 (2.0)	NYN†	33 (64.7)
	Y184**F**	14 (27.5)	N**F**N	13 (25.5)
	N1042**D**	4 (7.8)	NY**D**	3 (5.9)
			**Y**YN	1 (2.0)
			N**FD**	1 (2.0)
PfATP6(89/226/438/465/710)	I89**T**	5 (9.8)	IIAEG†	43 (84.3)
	I226**V**	1 (2.0)	**T**IAEG	2 (3.9)
	A438**D**	3 (5.9)	**T**I**D**EG	2 (3.9)
	N465**S**	1 (2.0)	**T**IA**K**G	1 (2.0)
	E710**K**	1 (2.0)	II**D**EG	1 (2.0)
			I**V**AEG	1 (2.0)
			IIA**K**G	1 (2.0)
Pfmdr6*(**R1**/175/**R2**/353/**R3**/SIN/823)	Y175**S**	9 (17.7)	6 Y2L6+N†	0
	L353**W**	1 (2.0)	8 Y2L6+–	25 (49.0)
	N823–	45 (88.2)	8 S2L6+–	5 (9.8)
			8 S2L6+N	4 (7.8)
			9 Y2L6+–	4 (7.8)
			8 Y2L5––	3 (5.9)
			6 Y2L6+–	2 (3.9)
			10Y2L6+–	2 (3.9)
			6 Y2W6+–	1 (2.0)
			7 Y2L6+–	1 (2.0)
			8 Y2L6+N	1 (2.0)
			8 Y3L6+–	1 (2.0)
			11Y2L5−N	1 (2.0)
			12Y2L4+–	1 (2.0)
PfMT(30/286/496)	I30**N**	50 (98.0)	ISN†	1 (2.0)
	S286**C**	1 (2.0)	**N**S–	48 (94.1)
	N496–	48 (94.1)	**N**SN	1 (2.0)
			**NCN**	1 (2.0)

† denotes the reference haplotype in 3D7; Letters in bold indicate mutated amino acids; - indicates deletion of the residue. No. (%) indicates the number and percentage of isolates harboring these mutations or haplotypes.

* The mdr6 haplotypes: R1 repeats, number of N at positions 103–110; R2, number of NI at positions 267–270, R3, number of NIN at positions 717–734; the tri-peptide SIN at position 735–737 are shown as + (present) or – (deleted).

#### Pfcrt

The key determinant of CQ resistance K76T was ubiquitously present in all samples. Further, the A220S mutation almost reached fixation and was found in 50/51 parasite isolates (data not shown).

#### PfATP6

We obtained complete sequences of the *PfATP6* coding region in 51 samples and compared them with that from 3D7. Mutations were rare and sporadic with a total of five identified (Table 3). Three mutations have been described previously (I89T, A438D and N465S), and two were novel mutations (I226V and E710K). Except for the I89T and A438D mutations, which occurred in 5 (9.8%) and 3 (5.9%) isolates of the samples, respectively, the remaining mutations were only detected once in the samples. The L263E and S769N mutations, which have been proposed to confer artemisinin resistance [30], [31], were not detected in our samples. A total of seven haplotypes were detected; 43 (84.3%) were wild-type, whereas six and two samples contained single and two mutations, respectively.

#### Pfmdr6

Analysis of the coding region of the *pfmdr6* gene from 51 parasite samples revealed mutations at residues 175 and 353 (Table 3). The Y175S mutation was found in 9 (17.7%) isolates, whereas L353W mutation was only found in one sample. In addition, this gene harbored two deletions. The tri-peptide SIN corresponding to positions 735–737 in 3D7 was deleted in four isolates, while the N residue at residue 823 was deleted in 45 (88.2%) isolates. Other polymorphisms all occurred in repeat sequences. For the microsatellite sequence corresponding to positions 103–110 in 3D7 (referred to as repeat R1), the number of the amino acid N ranged from 6 (in 3D7) to 12, with 8 as the predominant one occurring in 39 (76.5%) isolates. 3D7 had two NI repeats at positions 267–270 (referred to as repeat R2). Whereas 50 (98.0%) isolates had two NI repeats, only one field isolate had three NI repeats. The third repeat sequence, referred to as repeat R3, contains NIN repeats at positions corresponding to 717–734 in 3D7. Whereas 46 (90.2%) isolates had 6 NIN repeats, 4 (7.8%) and 1 (2.0%) isolates had 5 and 4 NIN repeats, respectively. These polymorphisms gave rise to a total of 13 haplotypes (Table 3), but none of the field isolates had the same haplotype as 3D7. The most prevalent haplotype, which differs from 3D7 by 8 N residues in microsatellite repeats R1 and a codon 819 deletion, occurred in 25 (49.0%) parasite isolates.

#### PfMT

Sequencing of the *pfMT* gene detected a total of two amino acid substitutions at positions 30 and 286. The I30N mutation was found in all but one parasite isolate, whereas the S286C mutation was rare and found only in one isolate. In addition, the codon for amino acid N496 was deleted in the majority (94.1%) of the isolates. A total of four amino acid haplotypes were recognized with one (N30/286S/496–) accounting for 94.1% parasite isolates (Table 3). In comparison, all other haplotypes only occurred once in the samples.

### Correlation between Polymorphisms and *in vitro* Drug Sensitivities

**Figure 2:**
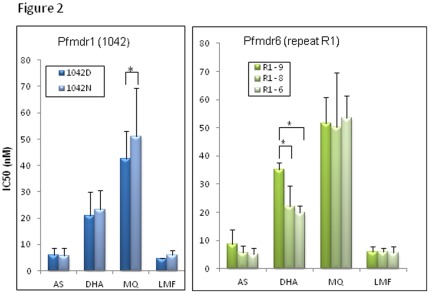
Association of SNPs and other polymorphisms in candidate genes with drug responses. Only significant association of a SNP in pfmdr1 and number variation of repeat R1 in pfmdr6 with *in vitro* responses to artesunate (AS), dihydroartemisinin (DHA), mefloquine (MQ) and lumefantrine (LMF) are plotted here. IC_50_ values are shown as mean + standard deviation. *indicates significant difference (*P*<0.05) in sensitivity between the two alleles.

Since the pfcrt K76T mutation was fixed and the A220S mutation almost reached fixation (50/51), and no *pfmdr1*amplification was detected in the studied parasite populations, associations could not be evaluated between these markers and drug responses. Thus, we analyzed the potential associations between point mutations in pfmdr1, pfATP6, pfmdr6, and pfMT genes and drug responses. None of the mutations in the pfmdr1 and pfATP6 genes had a significant association with altered responses to AS and DHA. Yet, the N1042D mutation in pfmdr1was found to be associated with increased susceptibility to MQ (Figure 2, Table S3). SNPs in *pfmdr6* and *pfMT* genes that caused amino acid substitutions were rare. None of them was associated with significantly altered sensitivity to the four drugs tested (*P*>0.05). Neither did the deletion mutations (S735−/I736−/N737– and N823– in pfmdr6, as well as N496– in pfMT) affect the sensitivity to the four drugs. There are three simple amino acid repeats in pfmdr6. Whereas no significant differences in drug sensitivity were detected between the parasites carrying different numbers of repeats in repeats R2 and R3, parasites carrying 9 repeats in repeat R1 were associated with significantly increased resistance to DHA (*P*<0.05) (Figure 2, Table S3). The difference between parasites carrying 8 and 9 R1 repeats remained significant after Bonferroni correction (Figure 2). In addition, 7 repeats in R1 were also significantly correlated with reduced sensitivity to LMF (P<0.05) (Table S3).

**Figure 3:**
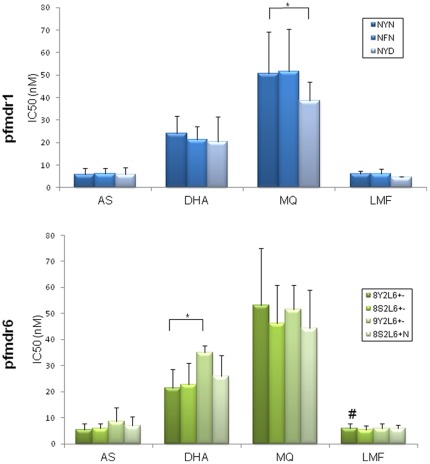
Association of haplotypes in candidate genes with drug responses. Only association of major haplotypes in pfmdr1 and pfmdr6 with *in vitro* responses to artesunate (AS), dihydroartemisinin (DHA), mefloquine (MQ) and lumefantrine (LMF) are plotted. IC_50_ values are shown as mean + standard deviation. * indicates significant difference (*P*<0.05) in sensitivity between the haplotypes after Bonferroni correction. # indicates that this pfmdr6 haplotype was significantly more sensitive to LMF than 3D7.

To determine whether certain haplotypes of *pfmdr1*, *pfATP6*, *pfmdr6*, and *pfMT* genes are associated with altered drug responses, we compared the drug responses of the mutant haplotypes with those of the wild-type isolates. Comparison of pfmdr1 haplotypes with ≥3 (5.9%) prevalence showed that parasites carrying the pfmdr1 1042D mutation showed increased sensitivity to MQ, whereas responses to other three drugs were not significantly different between the pfmdr1 haplotypes (Figure 3). All mutant haplotypes of pfATP6 were below 4% in prevalence, and only 2 (3.9%) parasite isolates with the 89T/438D double mutations had significantly higher IC_50_ values to AS (data not shown). For the *pfmdr6* gene, we found a significant association for the haplotype carrying 9 N residues in microsatellite repeat R1 (9Y2L6+–) with significantly higher DHA IC_50_ values when compared to both 3D7 and the haplotype 8Y2L6+–. These findings remained significant after Bonferroni correction (Figure 3). Furthermore, haplotype 8Y2L6+– showed significantly lower IC_50_s to LMF when compared to the wild-type 3D7. Interestingly, all of the haplotypes showed increased sensitivity to LMF when compared to 3D7. In comparison, none of the haplotypes in pfMT were associated with altered drug sensitivity (data not shown).

## Discussion

The GMS in Southeast Asia has been a breeding ground of antimalarial drug resistance, where MDR parasites have emerged. Parasites in this region appear to have the ARMD (accelerated resistance to multiple drugs) genetic background, favoring development of resistance to new antimalarial drugs [58], [59]. Further reflecting such a tendency, malaria parasites from this region have recently been found to exhibit reduced susceptibility or low-grade resistance to artemisinin derivatives [3], [4]. This urgent situation calls for closer surveillance, containment and heightened research on resistance mechanisms. In some areas of the GMS such as the China-Myanmar border area, artemisinins have been used for over three decades, mostly as monotherapies prior to 2005. Earlier *in vitro* assays have already detected a trend of declining sensitivity to AS [46]. Surveillance at the Thai-Myanmar border also detected reduced sensitivity to artemisinins, MQ, and QN [60]–[62]. Therefore, understanding whether artemisinin-resistant parasites have spread to neighboring regions or emerged elsewhere in the GMS is essential for coordinating containment efforts. Here, we evaluated this situation at the China-Myanmar border area and assessed the *in vitro* sensitivity of recently collected parasite isolates to artemisinins and two amino alcoholic drugs. Our data showed that although these parasites were generally sensitive to AS, there were eight parasite isolates showing mean IC_50_ values above 8 nM. In addition, *in vitro* sensitivity to DHA also had a more than 4-fold variation. Spearman’s correlation test detected significant correlations in sensitivity between the two artemisinin derivatives, suggesting that these drugs have shared cellular targets. Despite the fact that no careful clinical studies have been conducted in this region to rule out the lack of clinical resistance to artemisinins, our data suggest that reduced susceptibility to the artemisinins *might* have already emerged in some isolates (outliers) in the study parasite population. Therefore, close monitoring of resistance development at such sentinel sites is necessary. We have also included two aminoalcoholic drugs in the *in vitro* assays, since responses to MQ and LMF are often correlated with those to artemisinins [18], [61]. At the China-Myanmar border area, MQ has never been deployed, but *in vitro* assays suggested the existence of reduced sensitivity to MQ with 37 parasite isolates showing higher mean IC_50_ values than 3D7. Another perplexing finding is that the parasite isolates tested were highly sensitive to LMF, which is in sharp contrast to the parasites from Thailand that displayed more than 20-fold higher IC_50_s [63], although LMF has not been used in Thailand. The decreased sensitivity to LMF in Thailand could be related to the mass deployment of MQ in treating falciparum malaria, since sensitivities to MQ and LMF are correlated. Similarly, our samples collected in 2009 also showed significant correlation in IC_50_ values between these two drugs. However, none of these two aminoalcoholic drugs showed positive correlation in IC_50_ with the two artemisinin drugs. These are different from the *in vitro* drug studies carried out in other areas of the GMS [61], [64]–[66]. The reason for such discrepancies was not clear, but may be related to the divergent antimalarial drug policies in these areas.

While the mode of action of artemisinins is not completely understood, several cellular targets have been proposed. Among them, PfATP6 has been suggested as a prime candidate target of artemisinins [29], [30], and studies with field parasite isolates from French Guiana seemed to support this suggestion [31]. However, since these reports, results from subsequent studies appear to contradict the hypothesis of PfATP6 as a specific target of artemisinins. First, artemisinin treatment of *P. falciparum* did not alter the morphology of endoplasmic reticulum, where PfATP6 is supposed to be localized [67]. Second, purified recombinant PfATP6 did not seem to bind artemisinins, nor was it inhibited by artemisinins [68], [69]. Third, allelic exchange experiments aiming to determine whether the PfATP6 L283E mutation is linked to a drastic reduction of artemisinin sensitivity only provided tangential evidence supporting this suggestion [70]. In addition, our recent allelic exchange experiment did not detect differences in sensitivity to artemisinin derivatives between parasites carrying either S769 or 769N [71]. Further, an African parasite isolate carrying the S769N mutation did not show increased resistance to artemisinins [34]. In the Thai-Cambodian border region, where resistance to artemisinins has recently emerged, these two mutations have not been detected either [42]. We and others further confirmed the lack of these mutations in parasites from the China-Myanmar border area, where artemisinins have been deployed extensively for over 30 years [38]. In fact, most parasites from our study carried the wild-type *PfATP6* gene, and no significant association could be established between the rare mutations in *pfATP6* and artemisinin resistance. Collectively, these data potentially suggest that PfATP6 might not play a major and direct role in artemisinin resistance.

The malaria parasite encodes many transporters and some of them such as *pfcrt*, *pfmdr1* and *pfmrp* have been strongly connected with antimalarial drug resistance [72]–[74]. Parasite resistance to multiple drugs has been associated with SNPs and increased copy number of the ABC transporter family member *pfmdr1*. *Pfmdr1* amplification is correlated with resistance to MQ, QN, HF, and artemisinins [20], [75]. Laboratory experiments confirmed that an increase of *pfmdr1* copy number can be induced by stepwise MQ selection [76], whereas knockdown of *pfmdr1* expression results in increased susceptibility to MQ, QN, and artemisinin [77]. Moreover, recent laboratory selection studies strongly implicated *pfmdr1* gene amplification as a major determinant of induced artemisinin resistance [78], [79]. *Pfmdr1* also harbors many point mutations: the 86Y mutation which modifies resistance to CQ is associated with increased sensitivity to MQ and artemisinin [16], whereas mutations at the C-terminal end, which are prevalent in South America, contribute to QN resistance but enhance MQ and artemisinin sensitivity [18]. Consistently, administration of artemether-LMF has selected for increased frequency of the N86 allele [32], [80]. In Thailand and Cambodia, *pfmdr1* gene amplification has become increasingly prevalent in field parasite populations and was responsible for resistance to MQ and declining efficacy of ACTs [19], [22], [23], [26], [28]. In this case, *pfmdr1* amplification has been strongly tied to the extensive deployment of MQ in this region. At the China-Myanmar border, however, *pfmdr1* gene amplification has not been observed, consistent with the fact that MQ has never been used in this region. Overall, the frequencies of *pfmdr1* N86Y and C-terminal mutations which confer increased sensitivity to artemisinins were very low in the GMS [61], [64]. Our results from the China-Myanmar border further verified these findings [81]. Yet, despite the implied significance of *pfmdr1* in artemisinin resistance, in our study, *pfmdr1* was not associated with the reduced artemisinin susceptibility recently unveiled in western Cambodia [42]. The uncommon *pfmdr1* polymorphisms from these parasites offer opportunities for investigations of mechanisms other than *pfmdr1*.

The clear link between polymorphisms of transporter genes and antimalarial drug resistance has prompted us to evaluate other genes encoding putative transporters. We have chosen *pfmdr6*, another ABC transporter family member, and *pfMT*, a putative metabolite/drug transporter, since these two genes have been shown to be under recent positive selection from a genome-wide association study [43]. Both genes were found to harbor small numbers of nonsynonymous SNPs, whereas *pfmdr6* also have polymorphisms in three simple repeats. Compared with 3D7, the Y175S mutation and the 823 codon deletion had reached 17.7 and 88.2% of prevalence, respectively. However, none of the point mutations were associated with altered drug sensitivity. Yet, the microsatellite repeat R1, which has seven different alleles (ranging from 6 to 12 repeats), appeared to affect the *in vitro* sensitivity to DHA. Specifically, 9 Ns in R1 was associated with reduced sensitivity to DHA. This is reminiscent of the effect of the microsatellite repeats in the pfNHE1 gene on in vitro sensitivity to QN [50], [82]. Of the 13 haplotypes detected within this gene, 8 were rare haplotypes, each being represented by one or two isolates. Again, the haplotype with 9 Ns in R1 was associated with significantly higher IC_50_ value to DHA than others. Compared to 3D7, two mutations in pfMT (I30N and N496–) were predominant in the samples analyzed, which together gave rise to a predominant haplotype. Correlation studies did not detect any association of the mutations or haplotypes with significant altered drug sensitivity. However, we would like to exercise caution in this finding since previous *in vitro* studies of parasite resistance to AS have shown poor correlations with clinical *in vivo* studies. Furthermore, significant associations between these rare haplotypes and drug responses were at most based on a small number of parasite isolates carrying such mutations. Therefore, such an association may not hold if more samples carrying these mutations were analyzed.

Despite extensive efforts aimed at elucidating the mechanism of artemisinin resistance in *P. falciparum* using the candidate gene approach, most of the mutations encountered in candidate genes did not account for reduced sensitivity to artemisinins in parasites from clinical studies in western Cambodia [42]. Our *in vitro* studies using parasites from the China-Myanmar border area seem to convey a similar conclusion. However, analysis of parasites originating from the Thai-Myanmar border region implied that *pfmdr1* copy number and a new point mutation (F1226Y) are significantly associated with *in vitro* response to artemisinins [65]. This discrepancy may reflect the intrinsic difference between *in vitro* assay and *in vivo* clinical studies. In addition, it may also be due to divergent genetic backgrounds of the parasite populations, since the significance of *pfmdr1* point mutations for CQ and QN resistance appears to depend on the genetic background of the parasites [17], [18]. Meanwhile, another very relevant finding of this study is the existence of dramatic differences in drug responses and genotypes between our studied parasite population and other populations in the GMS. As malaria control is being intensified, geographical separation of parasite populations will occur. Differences in regional drug policy will inevitably exert distinctive selection pressures on parasites. Therefore, investigations into drug resistance mechanisms using different populations may help define the bona fide genetic determinants of artemisinin resistance. Furthermore, since artemisinin resistance appears to be a multigenic trait, mutations in multiple genes may have cumulative effects on parasite’s response to artemisinins. Consequently, genetic analysis at multiple loci and association studies with combined haplotypes may generate meaningful insights into the mechanisms of artemisinin resistance [65]. Genome-wide approaches such as SNP array and deep sequencing technologies may be crucial in identifying the molecular basis of the artemisinin resistance. It is also imperative that close resistance surveillance be continued at sentinel sites where artemisinins have been deployed extensively in the past so that the emergence and spread of artemisinin resistance is carefully monitored.

## Supporting Information

Figure S1 **Value of the** ***pfmdr1*** **copy number in 51** ***P. falciparum*** **isolates.** Each symbol indicates the *pfmdr1* copy number in one isolate.
(TIF)

Table S1 **Primers used in this study.**
(DOC)

Table S2 ***In vitro*** **IC_50_s (nM) of cultured field isolates to four antimalarial drugs** (mean ± standard deviation).
(DOC)

Table S3 ***In vitro*** **IC_50_s (nM) of parasite isolates to artesunate (AS), dihydroartemisinin (DHA), mefloquine (MQ) and lumefantrine (LMF) stratified by mutations in** ***pfmdr1*** **,** ***pfATP6*** **,** ***pfmdr6*** **and** ***pfMT*** **.**
(DOC)

## References

[1] Bosman A, Mendis KN (2007). A major transition in malaria treatment: the adoption and deployment of artemisinin-based combination therapies.. Am J Trop Med Hyg.

[2] White NJ (2008). Qinghaosu (artemisinin): the price of success.. Science.

[3] Dondorp AM, Nosten F, Yi P, Das D, Phyo AP (2009). Artemisinin resistance in *Plasmodium falciparum* malaria.. N Engl J Med.

[4] Noedl H, Se Y, Schaecher K, Smith BL, Socheat D (2008). Evidence of artemisinin-resistant malaria in western Cambodia.. N Engl J Med.

[5] Noedl H, Se Y, Sriwichai S, Schaecher K, Teja-Isavadharm P (2010). Artemisinin resistance in Cambodia: a clinical trial designed to address an emerging problem in Southeast Asia.. Clin Infect Dis.

[6] Wellems TE, Plowe CV (2001). Chloroquine-resistant malaria.. J Infect Dis.

[7] Roper C, Pearce R, Nair S, Sharp B, Nosten F (2004). Intercontinental spread of pyrimethamine-resistant malaria.. Science.

[8] Noedl H, Socheat D, Satimai W (2009). Artemisinin-resistant malaria in Asia.. N Engl J Med.

[9] Maude RJ, Pontavornpinyo W, Saralamba S, Aguas R, Yeung S (2009). The last man standing is the most resistant: eliminating artemisinin-resistant malaria in Cambodia.. Malar J.

[10] WHO (2009). Development of a strategy towards elimination of *Plasmodium falciparum* parasites with altered responses to artemisinins..

[11] Zarocostas J (2010). Better surveillance is needed to halt spread of artemisinin resistant malaria.. Bmj.

[12] Meshnick SR (2002). Artemisinin: mechanisms of action, resistance and toxicity.. Int J Parasitol.

[13] Krishna S, Woodrow CJ, Staines HM, Haynes RK, Mercereau-Puijalon O (2006). Re-evaluation of how artemisinins work in light of emerging evidence of in vitro resistance.. Trends Mol Med.

[14] Cui L, Su XZ (2009). Discovery, mechanisms of action and combination therapy of artemisinin.. Expert Rev Anti Infect Ther.

[15] Foote SJ, Kyle DE, Martin RK, Oduola AM, Forsyth K (1990). Several alleles of the multidrug-resistance gene are closely linked to chloroquine resistance in *Plasmodium falciparum*.. Nature.

[16] Duraisingh MT, Jones P, Sambou I, von Seidlein L, Pinder M (2000). The tyrosine-86 allele of the pfmdr1 gene of *Plasmodium falciparum* is associated with increased sensitivity to the anti-malarials mefloquine and artemisinin.. Mol Biochem Parasitol.

[17] Reed MB, Saliba KJ, Caruana SR, Kirk K, Cowman AF (2000). Pgh1 modulates sensitivity and resistance to multiple antimalarials in *Plasmodium falciparum*.. Nature.

[18] Sidhu AB, Valderramos SG, Fidock DA (2005). pfmdr1 mutations contribute to quinine resistance and enhance mefloquine and artemisinin sensitivity in *Plasmodium falciparum*.. Mol Microbiol.

[19] Pickard AL, Wongsrichanalai C, Purfield A, Kamwendo D, Emery K (2003). Resistance to antimalarials in Southeast Asia and genetic polymorphisms in pfmdr1.. Antimicrob Agents Chemother.

[20] Wilson CM, Volkman SK, Thaithong S, Martin RK, Kyle DE (1993). Amplification of pfmdr 1 associated with mefloquine and halofantrine resistance in *Plasmodium falciparum* from Thailand.. Mol Biochem Parasitol.

[21] Cowman AF, Galatis D, Thompson JK (1994). Selection for mefloquine resistance in *Plasmodium falciparum* is linked to amplification of the pfmdr1 gene and cross-resistance to halofantrine and quinine.. Proc Natl Acad Sci U S A.

[22] Price RN, Cassar C, Brockman A, Duraisingh M, van Vugt M (1999). The pfmdr1 gene is associated with a multidrug-resistant phenotype in *Plasmodium falciparum* from the western border of Thailand.. Antimicrob Agents Chemother.

[23] Price RN, Uhlemann AC, Brockman A, McGready R, Ashley E (2004). Mefloquine resistance in *Plasmodium falciparum* and increased pfmdr1 gene copy number.. Lancet.

[24] Alker AP, Lim P, Sem R, Shah NK, Yi P (2007). Pfmdr1 and in vivo resistance to artesunate-mefloquine in falciparum malaria on the Cambodian-Thai border.. Am J Trop Med Hyg.

[25] Shah NK, Alker AP, Sem R, Susanti AI, Muth S (2008). Molecular surveillance for multidrug-resistant *Plasmodium falciparum*, Cambodia.. Emerg Infect Dis.

[26] Wongsrichanalai C, Meshnick SR (2008). Declining artesunate-mefloquine efficacy against falciparum malaria on the Cambodia-Thailand border.. Emerg Infect Dis.

[27] Price RN, Uhlemann AC, van Vugt M, Brockman A, Hutagalung R (2006). Molecular and pharmacological determinants of the therapeutic response to artemether-lumefantrine in multidrug-resistant *Plasmodium falciparum* malaria.. Clin Infect Dis.

[28] Lim P, Alker AP, Khim N, Shah NK, Incardona S (2009). Pfmdr1 copy number and arteminisin derivatives combination therapy failure in falciparum malaria in Cambodia.. Malar J.

[29] Eckstein-Ludwig U, Webb RJ, Van Goethem ID, East JM, Lee AG (2003). Artemisinins target the SERCA of *Plasmodium falciparum*.. Nature.

[30] Uhlemann AC, Cameron A, Eckstein-Ludwig U, Fischbarg J, Iserovich P (2005). A single amino acid residue can determine the sensitivity of SERCAs to artemisinins.. Nat Struct Mol Biol.

[31] Jambou R, Legrand E, Niang M, Khim N, Lim P (2005). Resistance of *Plasmodium falciparum* field isolates to in-vitro artemether and point mutations of the SERCA-type PfATPase6.. Lancet.

[32] Happi CT, Gbotosho GO, Folarin OA, Sowunmi A, Hudson T (2009). Selection of *Plasmodium falciparum* multidrug resistance gene 1 alleles in asexual stages and gametocytes by artemether-lumefantrine in Nigerian children with uncomplicated falciparum malaria.. Antimicrob Agents Chemother.

[33] Dahlstrom S, Veiga MI, Ferreira P, Martensson A, Kaneko A (2008). Diversity of the sarco/endoplasmic reticulum Ca(2+)-ATPase orthologue of *Plasmodium falciparum* (PfATP6).. Infect Genet Evol.

[34] Cojean S, Hubert V, Le Bras J, Durand R (2006). Resistance to dihydroartemisinin.. Emerg Infect Dis.

[35] Mugittu K, Genton B, Mshinda H, Beck HP (2006). Molecular monitoring of *Plasmodium falciparum* resistance to artemisinin in Tanzania.. Malar J.

[36] Ferreira ID, Lopes D, Martinelli A, Ferreira C, do Rosario VE (2007). In vitro assessment of artesunate, artemether and amodiaquine susceptibility and molecular analysis of putative resistance-associated mutations of *Plasmodium falciparum* from Sao Tome and Principe.. Trop Med Int Health.

[37] Ibrahim ML, Khim N, Adam HH, Ariey F, Duchemin JB (2009). Polymorphism of PfATPase in Niger: detection of three new point mutations.. Malar J.

[38] Zhang G, Guan Y, Zheng B, Wu S, Tang L (2008). No PfATPase6 S769N mutation found in *Plasmodium falciparum* isolates from China.. Malar J.

[39] Tanabe K, Zakeri S, Palacpac NM, Afsharpad M, Randrianarivelojosia M (2011). Spontaneous Mutations in the *Plasmodium falciparum* Sarcoplasmic/Endoplasmic Reticulum Ca2+-ATPase (PfATP6) Gene among Geographically Widespread Parasite Populations Unexposed to Artemisinin-Based Combination Therapies.. Antimicrob Agents Chemother.

[40] Bacon DJ, McCollum AM, Griffing SM, Salas C, Soberon V (2009). Dynamics of malaria drug resistance patterns in the Amazon basin region following changes in Peruvian national treatment policy for uncomplicated malaria.. Antimicrob Agents Chemother.

[41] Hunt P, Afonso A, Creasey A, Culleton R, Sidhu AB (2007). Gene encoding a deubiquitinating enzyme is mutated in artesunate- and chloroquine-resistant rodent malaria parasites.. Mol Microbiol.

[42] Imwong M, Dondorp AM, Nosten F, Yi P, Mungthin M (2010). Exploring the contribution of candidate genes to artemisinin resistance in *Plasmodium falciparum*.. Antimicrob Agents Chemother.

[43] Mu J, Myers RA, Jiang H, Liu S, Ricklefs S (2010). *Plasmodium falciparum* genome-wide scans for positive selection, recombination hot spots and resistance to antimalarial drugs.. Nat Genet.

[44] Cui L, Yan G, Sattabongkot J, Cao Y, Chen B (2012). Malaria in the Greater Mekong Subregion: Heterogeneity and complexity.. Acta Trop 121.

[45] Delacollette C, D’Souza C, Christophel E, Thimasarn K, Abdur R (2009). Malaria trends and challenges in the Greater Mekong Subregion.. Southeast Asian J Trop Med Public Health.

[46] Yang H, Liu D, Yang Y, Fan B, Yang P (2003). Changes in susceptibility of *Plasmodium falciparum* to artesunate in vitro in Yunnan Province, China.. Trans R Soc Trop Med Hyg.

[47] Kaneko O, Kimura M, Kawamoto F, Ferreira MU, Tanabe K (1997). *Plasmodium falciparum*: allelic variation in the merozoite surface protein 1 gene in wild isolates from southern Vietnam.. Exp Parasitol.

[48] Snounou G, Zhu X, Siripoon N, Jarra W, Thaithong S (1999). Biased distribution of msp1 and msp2 allelic variants in *Plasmodium falciparum* populations in Thailand.. Trans R Soc Trop Med Hyg.

[49] Roper C, Richardson W, Elhassan IM, Giha H, Hviid L (1998). Seasonal changes in the *Plasmodium falciparum* population in individuals and their relationship to clinical malaria: a longitudinal study in a Sudanese village.. Parasitology.

[50] Meng H, Zhang R, Yang H, Fan Q, Su X (2010). In vitro sensitivity of *Plasmodium falciparum* clinical isolates from the China-Myanmar border area to quinine and association with polymorphism in the Na+/H+ exchanger.. Antimicrob Agents Chemother.

[51] Trager W, Jensen JB (1976). Human malaria parasites in continuous culture.. Science.

[52] Smilkstein M, Sriwilaijaroen N, Kelly JX, Wilairat P, Riscoe M (2004). Simple and inexpensive fluorescence-based technique for high-throughput antimalarial drug screening.. Antimicrob Agents Chemother.

[53] Cui L, Fan Q, Li J (2002). The malaria parasite *Plasmodium falciparum* encodes members of the Puf RNA-binding protein family with conserved RNA binding activity.. Nucleic Acids Res.

[54] Meng H, Zhang R, Yang H, Fan Q, Su X (2010). In vitro Sensitivity of *Plasmodium falciparum* Clinical Isolates from the China-Myanmar Border Area to Quinine and Association with Polymorphism in the Na+/H+ Exchanger.. Antimicrob Agents Chemother.

[55] Jambou R, Martinelli A, Pinto J, Gribaldo S, Legrand E (2010). Geographic structuring of the *Plasmodium falciparum* sarco(endo)plasmic reticulum Ca2+ ATPase (PfSERCA) gene diversity.. PLoS One.

[56] Yang Z, Zhang Z, Sun X, Wan W, Cui L (2007). Molecular analysis of chloroquine resistance in *Plasmodium falciparum* in Yunnan Province, China.. Trop Med Int Health.

[57] Duraisingh MT, Drakeley CJ, Muller O, Bailey R, Snounou G (1997). Evidence for selection for the tyrosine-86 allele of the pfmdr 1 gene of *Plasmodium falciparum* by chloroquine and amodiaquine.. Parasitology 114 (Pt.

[58] Rathod PK, McErlean T, Lee PC (1997). Variations in frequencies of drug resistance in *Plasmodium falciparum*.. Proc Natl Acad Sci U S A.

[59] Beez D, Sanchez CP, Stein WD, Lanzer M (2011). Genetic predisposition favors the acquisition of stable artemisinin resistance in malaria parasites.. Antimicrob Agents Chemother.

[60] Huttinger F, Satimai W, Wernsdorfer G, Wiedermann U, Congpuong K (2010). Sensitivity to artemisinin, mefloquine and quinine of *Plasmodium falciparum* in northwestern Thailand.. Wien Klin Wochenschr.

[61] Chaijaroenkul W, Wisedpanichkij R, Na-Bangchang K (2010). Monitoring of in vitro susceptibilities and molecular markers of resistance of *Plasmodium falciparum* isolates from Thai-Myanmar border to chloroquine, quinine, mefloquine and artesunate.. Acta Trop.

[62] Carrara VI, Zwang J, Ashley EA, Price RN, Stepniewska K (2009). Changes in the treatment responses to artesunate-mefloquine on the northwestern border of Thailand during 13 years of continuous deployment.. PLoS One.

[63] Mungthin M, Khositnithikul R, Sitthichot N, Suwandittakul N, Wattanaveeradej V (2010). Association between the pfmdr1 gene and in vitro artemether and lumefantrine sensitivity in Thai isolates of *Plasmodium falciparum*.. Am J Trop Med Hyg.

[64] Lim P, Wongsrichanalai C, Chim P, Khim N, Kim S (2010). Decreased in vitro susceptibility of *Plasmodium falciparum* isolates to artesunate, mefloquine, chloroquine, and quinine in Cambodia from 2001 to 2007.. Antimicrob Agents Chemother.

[65] Veiga MI, Ferreira PE, Jornhagen L, Malmberg M, Kone A (2011). Novel polymorphisms in *Plasmodium falciparum* ABC transporter genes are associated with major ACT antimalarial drug resistance.. PLoS One.

[66] Phompradit P, Wisedpanichkij R, Muhamad P, Chaijaroenkul W, Na-Bangchang K (2011). Molecular analysis of pfatp6 and pfmdr1 polymorphisms and their association with in vitro sensitivity in *Plasmodium falciparum* isolates from the Thai-Myanmar border.. Acta Trop.

[67] del Pilar Crespo M, Avery TD, Hanssen E, Fox E, Robinson TV (2008). Artemisinin and a series of novel endoperoxide antimalarials exert early effects on digestive vacuole morphology.. Antimicrob Agents Chemother.

[68] Cardi D, Pozza A, Arnou B, Marchal E, Clausen JD (2010). Purified E255L mutant SERCA1a and purified PfATP6 are sensitive to SERCA-type inhibitors but insensitive to artemisinins.. J Biol Chem.

[69] Arnou B, Montigny C, Morth JP, Nissen P, Jaxel C (2011). The *Plasmodium falciparum* Ca2+-ATPase PfATP6: insensitive to artemisinin, but a potential drug target.. Biochem Soc Trans.

[70] Valderramos SG, Scanfeld D, Uhlemann AC, Fidock DA, Krishna S (2010). Investigations into the role of the *Plasmodium falciparum* SERCA (PfATP6) L263E mutation in artemisinin action and resistance.. Antimicrob Agents Chemother.

[71] Cui L, Wang Z, Jiang H, Parker D, Wang H (2012). Lack of Association of the S769N Mutation in *Plasmodium falciparum* SERCA (PfATP6) with Resistance to Artemisinins..

[72] Mu J, Ferdig MT, Feng X, Joy DA, Duan J (2003). Multiple transporters associated with malaria parasite responses to chloroquine and quinine.. Mol Microbiol.

[73] Sanchez CP, Dave A, Stein WD, Lanzer M (2010). Transporters as mediators of drug resistance in *Plasmodium falciparum*.. Int J Parasitol.

[74] Kavishe RA, Koenderink JB, McCall MB, Peters WH, Mulder B (2006). Short report: Severe *Plasmodium falciparum* malaria in Cameroon: associated with the glutathione S-transferase M1 null genotype.. Am J Trop Med Hyg.

[75] Duraisingh MT, Cowman AF (2005). Contribution of the pfmdr1 gene to antimalarial drug-resistance.. Acta Trop.

[76] Preechapornkul P, Imwong M, Chotivanich K, Pongtavornpinyo W, Dondorp AM (2009). *Plasmodium falciparum* pfmdr1 amplification, mefloquine resistance, and parasite fitness.. Antimicrob Agents Chemother.

[77] Sidhu AB, Uhlemann AC, Valderramos SG, Valderramos JC, Krishna S (2006). Decreasing pfmdr1 copy number in *Plasmodium falciparum* malaria heightens susceptibility to mefloquine, lumefantrine, halofantrine, quinine, and artemisinin.. J Infect Dis.

[78] Chen N, Chavchich M, Peters JM, Kyle DE, Gatton ML (2010). Deamplification of pfmdr1-containing amplicon on chromosome 5 in *Plasmodium falciparum* is associated with reduced resistance to artelinic acid in vitro.. Antimicrob Agents Chemother.

[79] Chavchich M, Gerena L, Peters J, Chen N, Cheng Q (2010). Role of pfmdr1 amplification and expression in induction of resistance to artemisinin derivatives in *Plasmodium falciparum*.. Antimicrob Agents Chemother.

[80] Sisowath C, Stromberg J, Martensson A, Msellem M, Obondo C (2005). In vivo selection of *Plasmodium falciparum* pfmdr1 86N coding alleles by artemether-lumefantrine (Coartem).. J Infect Dis.

[81] Yang Z, Li C, Miao M, Zhang Z, Sun X (2011). Multidrug-resistant genotypes of *Plasmodium falciparum*, Myanmar.. Emerg Infect Dis.

[82] Ferdig MT, Cooper RA, Mu J, Deng B, Joy DA (2004). Dissecting the loci of low-level quinine resistance in malaria parasites.. Mol Microbiol.

