# Correction: In Vitro Sensitivity of *Plasmodium falciparum* from China-Myanmar Border Area to Major ACT Drugs and Polymorphisms in Potential Target Genes

**DOI:** 10.1371/journal.pone.0118328

**Published:** 2015-03-05

**Authors:** The PLOS ONE Staff

Dr. Miao Miao is not included in the author byline. Dr. Miao should be listed as the eighth author and affiliated with the Department of Entomology, Pennsylvania State University, University Park, Pennsylvania, United States of America. The contributions of this author are as follows: performed the experiments.
