# Supplementary figures and images for: In Vitro Sensitivity of Plasmodium falciparum from China-Myanmar Border Area to Major ACT Drugs and Polymorphisms in Potential Target Genes
Source: PLoS One. 2012 May 31;7(5):e30927. doi: 10.1371/journal.pone.0030927 (PMC3365119; doi:10.1371/journal.pone.0030927)

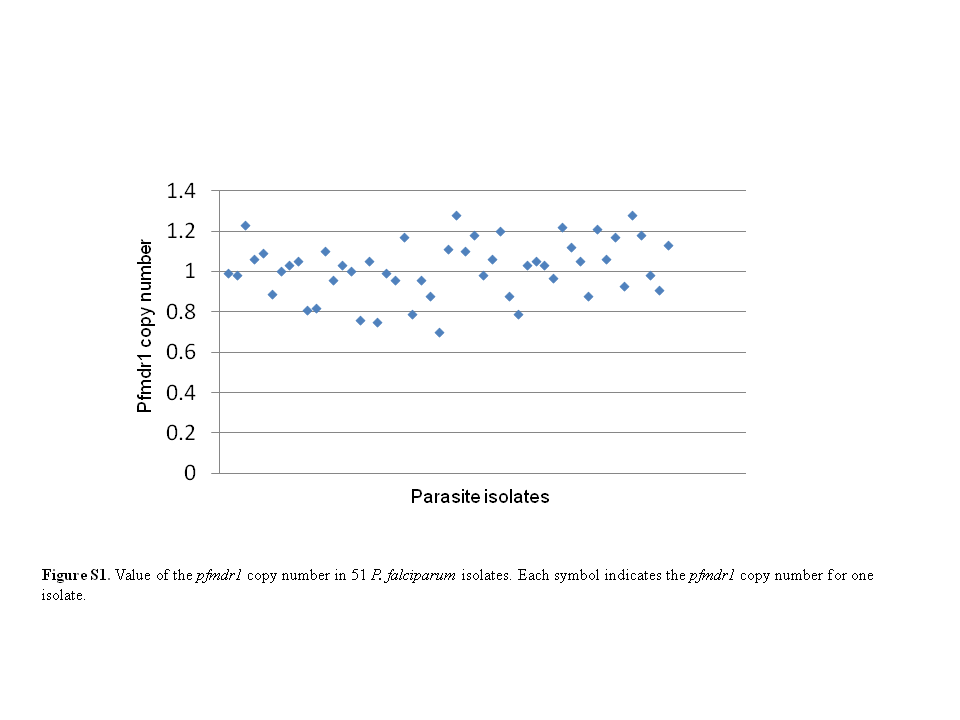

Supplement: Figure S1 — Value of the pfmdr1 copy number in 51 P. falciparum isolates. Each symbol indicates the pfmdr1 copy number in one isolate. (TIF) [file pone.0030927.s001.tif]
