# Supplementary material for: In Vitro Sensitivity of Plasmodium falciparum from China-Myanmar Border Area to Major ACT Drugs and Polymorphisms in Potential Target Genes
Source: PLoS One. 2012 May 31;7(5):e30927. doi: 10.1371/journal.pone.0030927 (PMC3365119; doi:10.1371/journal.pone.0030927)
Supplement: Table S1 — Primers used in this study. (DOC) [file pone.0030927.s002.doc]

**Table S1**. Primers used in this study.

| **Gene** | **Primer** | **Sequences** |
| --- | --- | --- |
| ***pfATP6*** | pfatpase6_1F | CATACGATGTTGAGGATGTAC |
|  | pfatpase6_2R | GACCTATTTCAGTCTTCATACC |
|  | pfatpase6_3F | GAGAATCCTGTTCAGTTGAC |
|  | pfatpase6_4R | ATCCTTCTTCTCCATCATCC |
|  | pfatpase6_5F | ACCGTGTTTCATTTGTTTAGAG |
|  | pfatpase6_6R | CCATTTGTTGTTGCCTGAGC |
|  | pfatpase6_7F | AATCACCAAGGGGTATCAAC |
|  | pfatpase6_8R | ACGTATACCAGCCATATGG |
|  | pfatpase6_9F | TTCAAAATATGGGAAAAAGAGCA |
|  | pfatpase6_10R | TGTGCTGGTAATCCGTCAG |
|  | pfatpase6-11F | TGCACCAGCATTGAAATCAG |
|  | pfatpase6_3039 | TACCTAGTGCTGTTGCTGGTAA |
|  | pfatpase6_3207 | AATCCACCAGAACATGACGTAAT |
|  | pfatpase6_3952 | TTTTCTTGGTTCTTTGCTCTTCC |
| ***Pfmdr6*** | ABC_43F | TCTGCACATTCATTATTTTGTCAAC |
|  | ABC_1786R | GCCCAAAATTTAGGAAAGACAA |
|  | ABC_1618F | GCGGAGCAAAATACATTCAA |
|  | mdr_1R | ATGATATTTATAAGAATTGTGAC |
|  | mdr_1764R | AGACTTTTGAACATTGATAGC |
|  | mdr_1648F | GATTCTATACAAAATGTCGAAC |
|  | mdr_2R | ATTGGAATATACAATAAGACAA |
|  | ABC_399F | TGACCTCAATGAAGCAAAAGAA |
|  | mdr_557R | TCTATACTTACATTATTATTCA |
|  | mdr_1120F | ACTCCGATATTATTATCCACA |
|  | mdr_2139R | GTTCGGATCCTTTTCTTTAGC |
|  | ABC_2527F | GGGAAAAGTGGTTCAGGAAAAAG |
|  | mdr_2643R | AGTATAAAGATCAATATCATC |
|  | ABC_3098R | TCATCCATGAGATTTTTGTGG |
| ***pfMT*** | mt_1F | CGGAGAAAGAGATAGAGGGA |
|  | mt_1R | CTTATTCTATATTTTTAACGTAACG |
|  | mt_2F | TATACTCCGTATTATTAAAGTAA |
|  | mt_2R | TAACCTATTAGGCTAAGTGGT |
|  | mt_412F | GATAGTGATTACAAAGAAACG |
|  | PF_14_260_1895R | GCACACAACCAGCTAACTGAA |
|  | mt_939R | GAAGATGGAAACAAAAACTGG |
|  | MT_17F | CAGCAAACAATGATGGTGAAA |
|  | mt_484R | TATCTTCCTCTTTGTAATTTGT |
|  | MT_873 | TGGATTTACTGGTTGGCGTA |
|  | mt_1433R | GATCTGCTATAACTTCTTCTGG |
|  | mt_1779F | GGGTATGTTGAATGGAATACC |
|  | MT_2356R | ACCACGGAAAACACGTAAGC |
