# Supplementary material for: In Vitro Sensitivity of Plasmodium falciparum from China-Myanmar Border Area to Major ACT Drugs and Polymorphisms in Potential Target Genes
Source: PLoS One. 2012 May 31;7(5):e30927. doi: 10.1371/journal.pone.0030927 (PMC3365119; doi:10.1371/journal.pone.0030927)
Supplement: Table S2 — In vitro IC50s (nM) of cultured field isolates to four antimalarial drugs (mean ± standard deviation). (DOC) [file pone.0030927.s003.doc]

**Table S2.** *In vitro* IC_50_s (nM) of cultured field isolates to four antimalarial drugs (mean ± standard deviation)

| Isolates | Mefloquine | Lumefantrine | Artesunate | Dihydroartemisinin |
| --- | --- | --- | --- | --- |
| f07-1 | 50.49±11.04 | 5.54±0.77 | 5.72±1.10 | 27.68±0.35 |
| f07-2 | 63.01±12.72 | 7.22±0.45 | 16.48±1.82 | 31.18±3.35 |
| f07-3 | 54.29±27.13 | 5.05±2.29 | 6.44±0.83 | 22.27±3.49 |
| f07-4 | 60.99±4.31 | 5.80±1.54 | 7.54±1.05 | 22.05±0.17 |
| f07-5 | 41.29±7.63 | 5.71±0.98 | 7.31±1.67 | 23.58±7.05 |
| f07-6 | 74.61±22.83 | 4.22±1.28 | 3.45±0.77 | 20.60±2.05 |
| f07-7 | 58.91±18.58 | 6.92±3.07 | 4.09±1.09 | 24.80±5.16 |
| f07-8 | 61.70±18.30 | 3.01±0.48 | 8.31±0.67 | 21.29±1.58 |
| f07-9 | 41.40±19.91 | 6.75±2.71 | 4.91±1.04 | 24.06±6.41 |
| f07-10 | 74.31±30.40 | 9.40±3.58 | 5.85±0.35 | 26.25±1.90 |
| f07-11 | 40.92±18.89 | 6.68±3.18 | 2.35±0.35 | 12.30±3.11 |
| f07-12 | 54.03±21.57 | 5.71±0.70 | 2.72±0.91 | 22.41±15.10 |
| f07-13 | 53.10±10.59 | 11.47±2.05 | 8.25±0.13 | 22.23±0.65 |
| f07-14 | 86.40±24.21 | 7.41±2.02 | 9.65±2.75 | 40.35±4.23 |
| f07-15 | 85.57±25.34 | 4.68±1.58 | 4.40±3.39 | 15.55±3.05 |
| f07-16 | 56.84±24.04 | 4.11±1.28 | 4.02±0.91 | 19.02±0.83 |
| f07-17 | 46.25±7.67 | 4.04±0.78 | 5.54±0.74 | 25.92±7.83 |
| f07-18 | 43.68±11.04 | 5.52±1.78 | 11.32±0.11 | 21.77±1.67 |
| f07-19 | 40.76±6.37 | 3.38±0.73 | 5.27±0.002 | 36.25±0.58 |
| f07-20 | 45.49±20.07 | 3.80±1.21 | 2.90±1.55 | 17.30±0.84 |
| f07-21 | 35.56±10.92 | 5.80±2.32 | 7.65±0.63 | 24.80±6.22 |
| f08-1 | 21.25±11.33 | 7.08±2.31 | 4.94±1.42 | 24.50±10.73 |
| f08-2 | 29.23±12.27 | 6.71±3.44 | 1.57±1.57 | 25.44±6.06 |
| f08-3 | 83.52±33.19 | 7.15±4.13 | 5.97±1.29 | 20.68±6.45 |
| f08-4 | 16.17±1.96 | 6.09±1.35 | 5.51±0.82 | 20.78±11.65 |
| f08-5 | 34.52±13.09 | 6.20±2.27 | 3.25±1.20 | 11.30±2.19 |
| f08-6 | 54.23±16.91 | 7.80±3.13 | 4.53±1.47 | 20.50±4.17 |
| f08-7 | 41.42±10.3 | 5.20±1.36 | 6.90±1.06 | 15.59±4.21 |
| f08-8 | 66.47±20.34 | 4.4±0.76 | 7.37±0.81 | 20.01±2.13 |
| f08-9 | 55.07±13.92 | 7.28±2.26 | 10.23±0.76 | 29.08±11.68 |
| f09-1 | 49.59±9.11 | 5.90±1.52 | 5.30±0.48 | 35.61±2.85 |
| f09-2 | 47.52±12.05 | 4.99±1.82 | 7.93±1.15 | 27.73±2.57 |
| f09-3 | 29.53±7.79 | 4.52±0.56 | 1.40±0.56 | 10.45±2.61 |
| f09-4 | 50.44±13.34 | 7.11±0.57 | 8.27±0.81 | 37.43±8.38 |
| f09-5 | 39.51±13.97 | 5.90±1.54 | 2.40±0.005 | 20.24±2.27 |
| f09-6 | 26.84±8.70 | 3.42±0.79 | 4.14±1.69 | 19.94±1.45 |
| f09-7 | 32.99±12.83 | 5.31±1.22 | 6.62±0.31 | 28.61±7. 91 |
| f09-8 | 34.53±15.64 | 4.47±2.19 | 9.36±3.86 | 33.29±4.84 |
| f09-9 | 32.84±15.39 | 4.34±1.82 | 3.88±1.62 | 14.25±0.89 |
| f09-10 | 67.26±15.65 | 4.18±0.79 | 7.80±0.20 | 25.00±2.00 |
| f09-11 | 48.24±15.47 | 4.9±0.99 | 3.99±1.24 | 13.25±4.92 |
| f09-12 | 57.73±16.95 | 5.52±2.14 | 1.75±0.35 | 19.75±1.06 |
| f09-13 | 49.06±22.75 | 8.11±2.76 | 6.27±0.45 | 16.74±8.06 |
| f09-14 | 96.24±18.87 | 8.22±2.60 | 2.60±1.13 | 11.50±3.67 |
| f09-15 | 43.71±11.06 | 4.31±1.94 | 4.55±0.74 | 22.21±0.24 |
| f09-16 | 53.48±14.65 | 3.61±1.95 | 5.26±0.78 | 9.55±0.10 |
| f09-17 | 34.23±11.65 | 5.41±2.33 | 4.56±0.95 | 26.00±9.56 |
| f09-18 | 86.47±27.13 | 8.71±4.43 | 7.40±0.80 | 18.50±2.82 |
| f09-19 | 24.88±7.35 | 4.11±2.11 | 4.10±1.85 | 20.35±9.40 |
| f09-20 | 54.37±32.30 | 7.27±3.11 | 7.17±2.38 | 37.21±8.94 |
| f09-21 | 39.24±10.37 | 8.21±1.05 | 6.88±1.09 | 35.92±0.22 |
