# Supplementary material for: In Vitro Sensitivity of Plasmodium falciparum from China-Myanmar Border Area to Major ACT Drugs and Polymorphisms in Potential Target Genes
Source: PLoS One. 2012 May 31;7(5):e30927. doi: 10.1371/journal.pone.0030927 (PMC3365119; doi:10.1371/journal.pone.0030927)
Supplement: Table S3 — In vitro IC50s (nM) of parasite isolates to artesunate (AS), dihydroartemisinin (DHA), mefloquine (MQ) and lumefantrine (LMF) stratified by mutations in pfmdr1 , pfATP6 , pfmdr6 and pfMT . (DOC) [file pone.0030927.s004.doc]

**Table S3.** *In vitro* IC_50_s (nM) of parasite isolates to artesunate (AS), dihydroartemisinin (DHA), mefloquine (MQ) and lumefantrine (LMF) stratified by point mutations in *pfmdr1*, *pfATP6*, *pfmdr6* and *pfMT*.¶

| **Gene** | **Amino acid** | | **No. of isolates (n=51)** | **AS** | **DHA** | **MQ** | **LMF** |
| --- | --- | --- | --- | --- | --- | --- | --- |
| **pfmdr1** | 86N | 50 | | 5.9 (2.8) | 23.1 (7.4) | 50.3 (18.0) | 5.9 (1.7) |
|  | 86**Y** | 1 | | 4.0 | 19.0 | 56.8 | 4.1 |
|  | 184Y | 37 | | 5.7 (3.0) | 23.6 (7.9) | 49.8 (17.9) | 5.8 (1.5) |
|  | 184**F** | 14 | | 6.2 (2.2) | 21.4 (5.7) | 51.8 (18.3) | 5.9 (2.3) |
|  | 1042N | 47 | | 5.8 (2.8) | 23.2 (7.3) | 51.1 (18.3) | 6.0 (1.8) |
|  | 1042**D** | 4 | | 5.9 (2.6) | 20.8 (9.3) | 42.5* (10.5) | 4.7 (0.3) |
| **pfATP6** | 89I | 46 | | 5.7 (2.8) | 22.8 (7.5) | 50.1 (18.6) | 5.9 (1.8) |
|  | 89**T** | 5 | | 6.4 (2.7) | 24.9 (7.1) | 53.1 (7.8) | 5.3 (1.5) |
|  | 226I | 50 | | 5.8 (2.8) | 23.0 (7.5) | 50.6 (18.0) | 5.8 (1.7) |
|  | 226**V** | 1 | | 4.9 | 24.1 | 41.4 | 6.8 |
|  | 438A | 48 | | 5.7 (2.8) | 23.1 (7.6) | 49.8 (18.2) | 5.9 (1.7) |
|  | 438**D** | 3 | | 7.4 (0.9) | 21.9 (0.5) | 59.0 (4.1) | 4.6 (1.4) |
|  | 465N | 50 | | 5.9 (2.7) | 23.1 (7.5) | 50.2 (18.0) | 5.9 (1.7) |
|  | 465**S** | 1 | | 1.8 | 19.8 | 57.7 | 5.5 |
|  | 710E | 50 | | 5.8 (2.8) | 22.9 (7.5) | 50.5 (18.0) | 5.9 (1.7) |
|  | 710**K** | 1 | | 5.5 | 25.9 | 46.3 | 4.0 |
| **pfmdr6^#^** | R1 - 6 | 3 | | 5.0 (2.4) | 20.0 (2.4) | 53.6 (7.8) | 5.8 (2.0) |
|  | R1 - **7** | 1 | | 8.3 | 22.2 | 53.1 | **11.5^§^** |
|  | R1 - **8** | 39 | | 5.7 (2.4) | 22.1**^+^** (7.3) | 50.3 (19.4) | 5.8**^§^** (1.5) |
|  | R1 - **9** | 4 | | 8.6 (5.4) | 35.1**^+^** (2.7) | 51.7 (9.2) | 5.9 (1.8) |
|  | R1 - **10** | 2 | | 2.9 (0.7) | 20.4 (0.3) | 57.1 (24.8) | 5.1**^§^** (1.2) |
|  | R1 - **11** | 1 | | 7.9 | 27.7 | 47.5 | 5.0 |
|  | R1 - **12** | 1 | | 4.1 | 19.9 | 26.8 | 3.4**^§^** |
|  | 175Y | 42 | | 5.7 (2.8) | 22.7 (7.4) | 51.4 (18.6) | 5.9 (1.8) |
|  | 175**S** | 9 | | 6.4 (2.6) | 24.3 (7.7) | 45.4 (13.7) | 5.6 (1.5) |
|  | R2 - 2 | 50 | | 5.8 (2.8) | 23.0 (7.5) | 50.5 (18.0) | 5.9 (1.7) |
|  | R2 - **3** | 1 | | 4.6 | 22.2 | 43.7 | 4.3 |
|  | 353L | 50 | | 5.9 (2.8) | 23.1 (7.4) | 50.5 (18.0) | 5.9 (1.7) |
|  | 353**W** | 1 | | 2.9 | 17.3 | 45.4 | 3.8 |
|  | R3 - 6 | 46 | | 5.8 (2.8) | 22.8 (7.6) | 51.1 (18.3) | 6.0 (1.8) |
|  | R3 - **4** | 1 | | 4.1 | 19.9 | 26.8 | 3.4 |
|  | R3 - **5** | 4 | | 6.4 (3.3) | 25.8 (6.0) | 48.5 (10.2) | 5.0 (0.4) |
|  | 735S/736I/737N | 47 | | 5.8 (2.8) | 22.8 (7.5) | 50.5 (18.4) | 5.9 (1.8) |
|  | 735–/736–/737– | 4 | | 6.4 (3.3) | 25.8 (6.0) | 48.5 (10.2) | 5.0 (0.4) |
|  | 823N | 6 | | 6.5 (3.2) | 23.9 (8.7) | 43.3 (12.1) | 5.8 (1.2) |
|  | 823**–** | 45 | | 5.7 (2.7) | 22.9 (7.3) | 51.3 (18.4) | 5.9 (1.8) |
| **pfMT** | 30I | 1 | | 5.8 | 23.0 | 41.4 | 5.8 |
|  | 30**N** | 50 | | 5.8 (2.8) | 23.0 (7.5) | 50.6 (18.0) | 5.8 (1.7) |
|  | 286S | 50 | | 5.8 (2.8) | 23.0 (7.5) | 50.7 (17.9) | 5.9 (1.7) |
|  | 286**C** | 1 | | 7.7 | 24.8 | 35.6 | 5.8 |
|  | 496N | 3 | | 7.0 (1.8) | 23.4 (1.9) | 46.2 (13.7) | 5.2 (1.9) |
|  | 496**–** | 48 | | 5.7 (2.8) | 23.0 (7.6) | 50.6 (18.2) | 5.9 (1.7) |

¶IC_50_s are shown as mean (standard deviation). Amino acids in bold indicate mutations as compared with the wild-type allele in 3D7.

***** Indicates statistical significance (*P*≤ 0.05) by the generalized linear model in IC_50_s between parasite carrying the wild-type and mutant alleles.

+ and § Indicate significant differences between polymorphisms using *t­*-tests and a Bonferroni correction. For those indicated with § the number in bold indicates the type that is significantly different than the others.

# The mdr6 polymorphisms: R1 repeats, number of N at positions 103-110; R2, number of NI at positions 267-270, R3, number of NIN at positions 717-734.
